# GPA33-Targeted Trimeric Immunotoxin Exhibits Enhanced Antitumor Activity in Human Colorectal Cancer Xenografts

**DOI:** 10.3390/ijms27020764

**Published:** 2026-01-12

**Authors:** Javier Ruiz-de-la-Herrán, Javier Narbona, Rubén G. Gordo, Laura Sanz, Javier Lacadena

**Affiliations:** 1 Department of Biochemistry and Molecular Biology, Faculty of Chemical Sciences, Complutense University, 28040 Madrid, Spain; chaosrorri@hotmail.com (J.R.-d.-l.-H.); jnarbona@ucm.es (J.N.); rugarc09@ucm.es (R.G.G.); 2 Molecular Immunology Unit, Biomedical Research Institute, Hospital Universitario Puerta de Hierro, Majadahonda, 28222 Madrid, Spain; lsalcober@salud.madrid.org

**Keywords:** immunotoxin, trimeric, antibody engineering, colorectal cancer, antitumor efficacy, α-sarcin

## Abstract

Immunotoxins are chimeric molecules with high potential as therapeutic candidates that combine antibody specificity to recognize and bind tumor-associated antigens and the cytotoxic potency of the enzymatic activity of a toxin, leading to the selective death of target cells. The use of immunotoxins as therapeutic tools remains limited by various issues, such as selecting the appropriate tumor-associated antigen (TAA), penetration difficulties in solid tumors, low renal clearance, and low toxic payload. For this purpose, in this work we have designed a novel trimeric immunotoxin (IMTXTriA33αS) against colorectal cancer, combining the scFv against GPA33 as a targeting domain and the fungal ribotoxin α-sarcin (αS) as the toxic fragment, linked by a trimerization domain (TIE^XVIII^). Our results demonstrate that IMTXTriA33αS has greater avidity and toxic load, showing a very significant increase in its in vitro and in vivo antitumor efficacy, due to its trimeric structure.

## 1. Introduction

Antibody-based immunotherapy is one of the strategies used in the treatment of different types of cancer, and inside this area the application of immunotoxins is taking relevance. Immunotoxins (IMTXs) are chimeric proteins composed of a targeting domain fused to a toxic domain that causes specifically the death of the target cells. The use of IMTXs in cancer therapy is an emergent field of study nowadays [1,2,3], with three approved constructions for the treatment of cancer: Denileukin difititox, targeting IL2R and the *Diphteria* toxin (DT) as the toxic domain; Tagraxofusp, also with DT as the toxic domain but targeting IL3R instead; and Moxetumomab pasudotox, targeting CD22 and with *Pseudomonas* exotoxin A as a toxic moiety [4,5]. Usually, the targeting domain includes a full-length monoclonal antibody (mAb), or an mAb-derived structure, such as Fab fragments or single-chain variable domains (scFv). More recently, other immunotoxins have been described including nanobodies or variable regions of the heavy chain of camelid antibodies (V_HH_) in their signaling domain, as well as other types of molecules, such as cytokines, like IL2 [6].

Regarding the toxic domain, several toxins from different origins have already been used to generate IMTXs. Thus, designs based on plant toxins, such as gelonin and ricin, have been described, both exerting their action against rRNA 28s by different mechanisms [6]. These toxins, usually considered as ribosome inactivating proteins (RIPs) have been considerably improved regarding their stability [7] and antitumoral effect [8]. Alternatively, toxins from bacteria have been used, like *Pseudomonas* exotoxin A or *Corynebacterium* diphtheria toxin, that inhibit protein biosynthesis through ADP-ribosylation of eukaryotic elongation factor-2 (eEF2). Further, fungal toxins were also included as the toxic domain, with α-sarcin standing out as the most relevant member of the fungal ribotoxin family. Its small size, high ribonucleolytic specificity, high thermal and protease resistance, and low immunogenicity have made it an ideal candidate for immunotoxin designs [9,10].

Different types of IMTXs targeted against colorectal cancer and based on α-sarcin have been described and characterized, such as a monomeric GPA33 (glycoprotein A33)-target IMTX, monomeric and trimeric anti-CEA IMTXs [11], and EGFR-targeted IMTXs [12], exhibiting high specificity and potent cytotoxic effects on tumor cells. The intracellular pathway of these IMTXs after internalization has also been studied. In this sense, α-sarcin-based IMTXs, after binding to the antigen on the cell surface, enter the cell by endocytosis followed by Golgi apparatus colocalization [13,14]. The toxic domain then translocates into the cytosol from the endosomes or from the Golgi apparatus, due to its ability to interact with acid phospholipids, with Golgi apparatus having a negatively charged bilayer.

GPA33 is a specific marker of colorectal cancer cells expressed on 95% of colorectal cancers [15,16]. Due to this specificity in tumor cells, GPA33 is an interesting target for α-sarcin-based IMTX in comparison with other tumor-associated antigens. For example, CEA shedding, due to proteolytic cleavage, may decrease the effectiveness of an anti-CEA-based IMTX. Instead, GPA33 remains anchored in the membrane, without being secreted [17,18], preventing the loss of treatment efficiency. Moreover, GPA33 exhibits a much lower expression profile in healthy tissues than EGFR, consequently reducing possible off-target toxicities. Thus, GPA33 would constitute a more suitable TAA for targeting than EGFR [19]. GPA33 on tumor cells has a function related to the maintenance of the mucosa produced by the gut and to the regulation of the intestinal immune response [20]. Furthermore, it has been described that GPA33 is involved in adhesion and intracellular trafficking processes [21,22,23,24,25], and its expression is mediated by GKLF (gut-enriched Krüppel-like factor), and thus related with proliferation and growth cell function [24,26]. Moreover, its expression is related to the step of the cellular cycle and regulated by intracellular trafficking, which could explain its expression on most colorectal tumors, with this expression being totally polarized to the basolateral region of the gut, reaching the amount of 800,000 molecules per cell [23,27].

These properties all together make GPA33 a good target for cancer treatment. A humanized monoclonal antibody against GPA33 and scFvA33 fragments derived of it have been described [28]. The scFvA33 fragments can be expressed and correctly folded by the yeast *Pichia pastoris* [29,30], and have a better penetration in solid tumors than the whole antibody.

Using a modular approach, recombinant antibodies can be easily engineered into diverse and improved formats based on antibody fragments, including scFvs or V_HH_ [31], allowing for deeper tumor penetration [12,32], the recruitment and redirection of T cells towards tumor antigens including an anti-CD3 moiety in T cell engagers (TCEs) [33,34], and/or the simultaneous targeting of different TAAs on the same tumor, preventing the decrease in the therapeutic effect due to antigen loss or selective mutations [35]. Interestingly, trimerization domains can also be added to constitute multimeric moieties.

Some studies revealed that dimeric IMTXs have more effectiveness against tumor cells than the monomeric ones, leading to the exploration of the possible multimeric IMTXs. It has been reported that trimerbodies, based on the human collagen XVIII-derived trimerization domain (TIE^XVIII^) fused into the N- and/or C-terminal region of the binding domain [36,37], have enhanced functional affinity compared to their monomeric counterparts [38]. Due to their size, trimerbodies have a reduced renal clearance, an increased half-life, and also show an enhanced avidity towards the antigen, leading to an increased uptake into tumor cells that expressed the target protein or receptor [39,40]. These trimeric proteins adopt a pyramidal structure stabilized by interactions between the TIE^XVIII^ domains of each monomer, which has been described previously for human collagen XVIII [41]. This strategy has been used in the development of trimerbodies using scFv or V_HH_ fragments. [42,43].

The first trimeric IMTX using the TIE^XVIII^ domain was directed against the CEA antigen, with positive results and improved characteristics in comparison with its monomeric form, but with problems related to CEA shedding [11]. In addition, a trimeric nanoIMTX, whose target domain consisted of an anti-EGFR V_HH_, exhibited increased binding and antitumor effects in vitro compared to the monomeric version [12]. Thus, this trimeric IMTX using the scFv against GPA33 as the target domain should be a good strategy to treat cancer, regarding its effectiveness on a monomeric form, and with the enhanced effectiveness of a trimeric design.

We have previously described the potent antitumor effect in vitro [44] and in vivo of the original monomeric immunotoxin (IMTXA33αS) [45]. Because of the potential advantages of the trimeric format, and the previous results of the monomeric IMTXA33αS, in this work we have designed, produced, and characterized a new trimeric IMTX against GPA33, including α-sarcin as the toxic domain. The results obtained for the structural and functional characterization of IMTXTriA33αS showed an increase in its antitumor efficacy both in vitro and in vivo, suggesting promising therapeutic potential. The results presented here constitute a new step for the potential therapeutic application of α-sarcin-based immunotoxins combining different formats in terms of structure and specificity.

## 2. Results

### 2.1. Trimeric IMTX Design, Expression, and Purification

**Figure 1:**
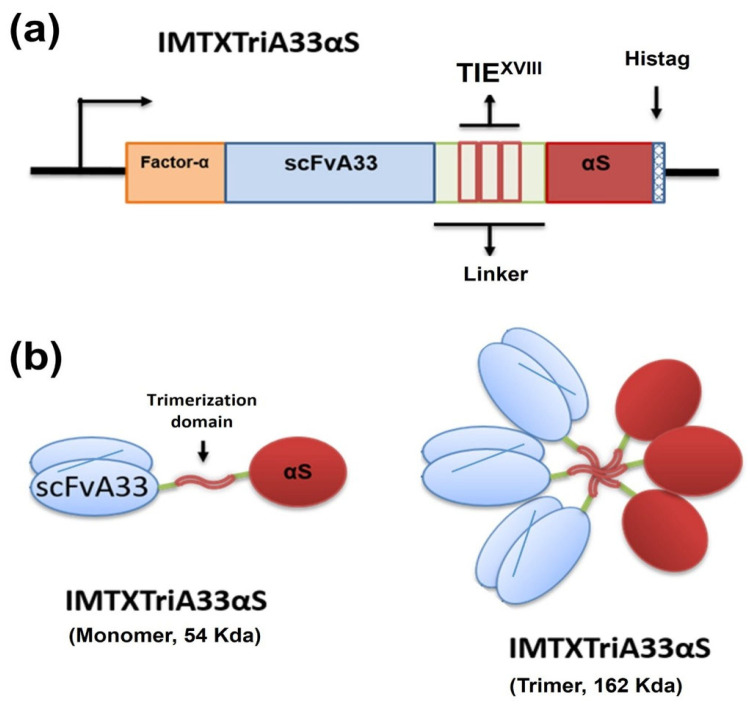
Schematic diagrams showing the genetic and domain arrangements of immunotoxin design: IMTXTriA33αS. (**a**) The cDNA construct employed. (**b**) The schematic representation of the native proteins displaying their different domain arrangements. In both cases, structural and functional motifs are highlighted with colors: α-factor secretion signal peptide (orange); scFvA33 (light blue); L21 linkers (light green); TIE^XVIII^, human non-colagenous trimerization domain (TIE, light pink); α-sarcin (red); and histidine-tag (blue X). Native IMTXTriA33αS should display a theoretical size of 162 kDa; meanwhile, for monomeric IMTXA33αS, the expected mass value should be 54 kDa.

The vector designed to express IMTXTriA33αS (pPICZαATriA33αS) (Figure 1) was obtained using molecular biology techniques, confirmed through Sanger sequencing by the Genomic Unit at the University Complutense of Madrid, and finally electroporated into Bg11 *P. pastoris* cells.

**Figure 2:**
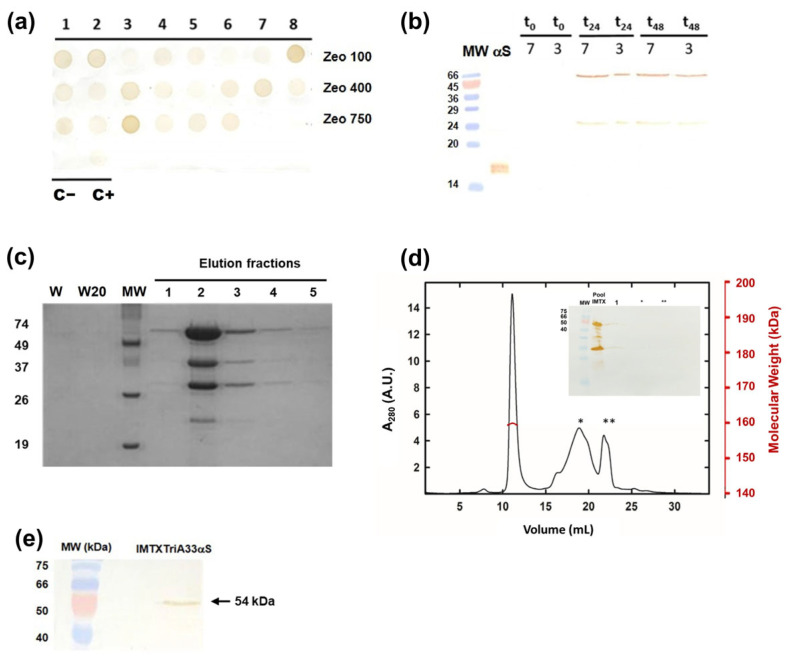
Production and purification assays. (**a**) Multiple colony expression analysis by Dot blot. The numbers indicate the colony number among those tested for each zeocin concentration. C− and C+, located in the 4th row, correspond to the negative control (non-transformed colonies) and positive control (IMTXA33αS-producing colonies), respectively. (**b**) Western blot analysis of expression assays of 3-Zeo750 and 7-Zeo400 colonies at different induction times (t0, t24, and t48). MW corresponds to the molecular mass pattern. The second lane corresponds to α-sarcin control. (**c**) Coomassie Blue-stained SDS-PAGE analysis of different aliquots taken during affinity chromatography. Lines correspond to the following: W, washed fraction eluted with chromatography buffer; W20, eluted with the chromatography buffer containing 20 mM imidazole; and Elution fractions, the first five 1 ml fractions eluted with 250mM imidazole. (**d**) Superdex 200 FPLC gel filtration analysis of IMTXTriA33αS. Major peak corresponds to oligomeric IMTXTriA33αS weight (red axis). Peaks marked with * or ** correspond with degradation products of the immunotoxin. Inset: SDS-PAGE analysis of SEC-FPLC peaks. (**e**) Western blot analysis of purified IMTXTriA33αS using rabbit anti-α-sarcin serum. MW corresponds to prestained Bio-Rad Precision Plus protein standards. Images corresponding to full-length gels and blots were acquired and analyzed using the Gel Doc XR Imaging System and Quantity One 1-D v4.6.9. analysis software (BioRad) or ChemiDoc-It (UVP) and VisionWorks LS, respectively. Original full-length blots are presented in Supplementary Figure S1.

The electroporation mixture was seeded in a tissue culture disk with culture media containing different concentrations of Zeocine for selecting the colonies with higher levels of IMTX expression. With this objective, several expression assays were performed. In a first step, Dot blot screening using an anti-α-sarcin antibody allowed us to select two of the colonies, 3-Zeo^750^ and 7-Zeo^400^ (Figure 2a), to perform a low scale production of the colonies. Next, the expression level of both colonies at 24/48 h was determined by Western blot, with an increased expression of IMTXTriA33αS observed for 7-Zeo^400^ (Figure 2b).

The IMTXTriA33αS was purified from the extracellular medium as previously described for other IMTXs by Ni^2+^-NTA affinity chromatography [44,45,46]. The different purifications steps were analyzed by PAGE-SDS (Figure 2c–e). Fractions containing IMTXTriA33αS were dialyzed together on 25 kDa pore membranes, for the elimination of imidazole and degrading products. An additional purification step using size exclusion chromatography (SEC-FPLC) was carried out not only to eliminate the degraded protein but also for structural characterization (Figure 2d). The 54 kDa size band corresponds to the monomer of the IMTX. In addition, other bands corresponding to degraded forms of the monomer were also observed. The extinction coefficient (ɛ: 78,770 M^−1^_·_cm^−1^) was determined by absorption spectroscopy and amino acid analysis and was used to determine the concentrations of the IMTX for the following assays. The purification yield was around 1 mg of IMTX per liter of induction media, which represented a similar purification yield compared to other IMTXs previously produced.

### 2.2. Structural Characterization

#### 2.2.1. Oligomerization Study (FPLC)

The presence of the trimeric form in the solution of native IMTXTriA33αS was studied by SEC-FPLC. The elution profile of the Superdex 200 10/300 GL showed the presence of various absorption peaks corresponding to the different oligomerization states of the IMTX. The eluted proteins showed a single symmetric elution major peak at the expected volume for the predicted 160 kDa for the trimeric form of IMTXTriA33αS (Figure 2d). The other peaks correspond to the monomeric IMTX and degraded forms of it. The major peak fractions (Figure 2e) were collected for functional characterization.

#### 2.2.2. Secondary Structure Analysis (Circular Dichroism)

**Figure 3:**
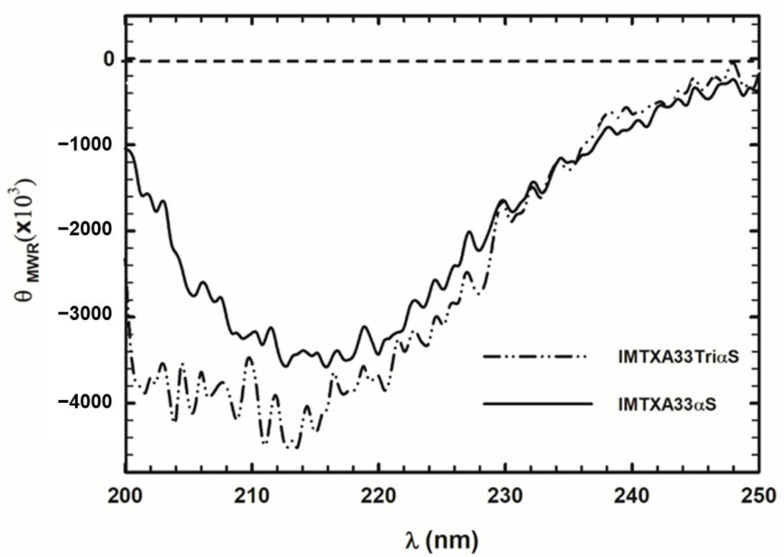
Structural characterization. Far-UV CD spectra of IMTXTriA33αS (dashed-dotted line) and IMTXA33αS (solid line) are shown (θ_MRW_, mean residue weight ellipticities expressed as degree × cm^2^ × dmol^−1^). Both spectra were performed in 50 mM sodium phosphate, 0.1 M NaCl pH 7.4 at a protein concentration of 0.15 mg/mL.

To corroborate the correct folding of IMTXTriA33αS, a far-UV circular dichroism (CD) spectrum was obtained and compared to the one of the original IMTXA33αS. As shown in Figure 3, the CD spectrum of the trimeric IMTX was compatible with globular folded proteins, related to the high contribution of the β-sheet secondary structure, with coherence with previous studies of the toxic and targeting domains (α-sarcin and scFvA33) [44]. The differences observed on the spectra between IMTXA33αS and IMTXTriA33αS suggest an increase in the random secondary structure, as a consequence of the presence of the trimerization domain and the linker loops [12,36].

### 2.3. Functional Characterization

#### 2.3.1. Ribonuclease Activity

**Figure 4:**
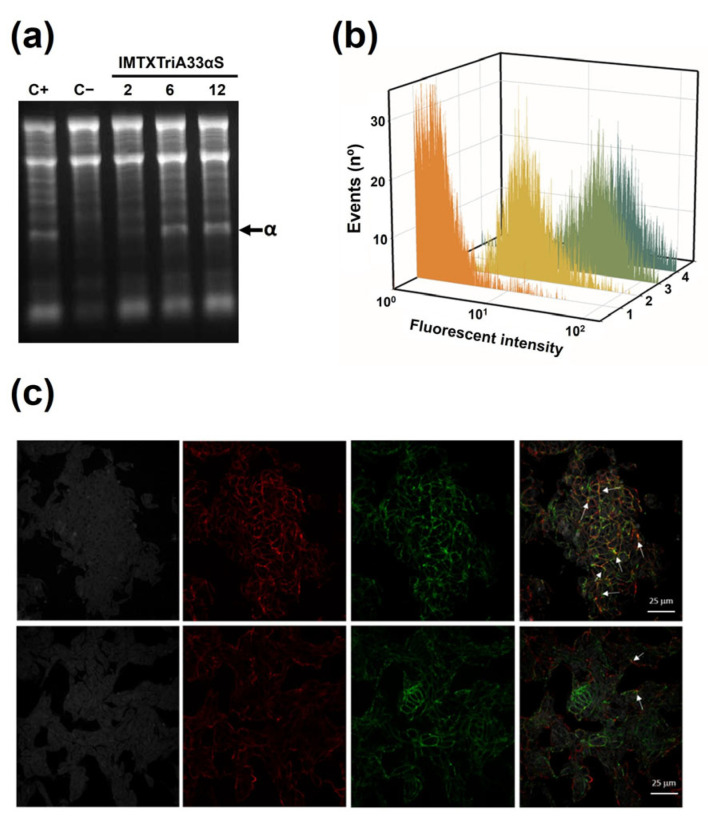
In vitro functional characterization. (**a**) Rabbit reticulocytes assay was carried out to assay the ribonucleolytic activity of α-sarcin in IMTXTriA33αS. The gel shows the release of α-fragment (α, highlighted by a black arrow), produced by the specific SRL cleavage. Measurements of 2, 6, and 12 pmol were assayed for the immunotoxin and 6 pmol for fungal wild-type α-sarcin. C−, negative control contains buffer instead of protein. Images correspond to full-length gels acquired and were analyzed using the Gel Doc XR Imaging System and Quantity One 1-D analysis software (BioRad). Original full-length gel is presented in Supplementary Figure S2. (**b**) Binding assay of the targeting domain by flow cytometry analysis with SW1222 (GPA33-positive) cells. Curves correspond to cells incubated with (1) secondary antibody anti-His-Alexa-488, (2) IMTXA33αS 0.1 mM, and IMTXTriA33αS 0.01 mM (3) or 0.1 mM (4). Fluorescence intensity was represented in arbitrary but proportional units. (**c**) Confocal microscopy images of SW1222 cells incubated with IMTXTriA33αS for 20 min (top row) or 1 h (bottom row). Images correspond, from left to right, to nuclei (DAPI, gray), plasma membrane (CD44, red), IMTXTriA33αS-555 (green), and overlay of the previous images. In the latter, yellow color indicates colocalization at the plasma membrane.

To carry out the IMTX functional characterization, the specific ribonucleolytic activity of α-sarcin was first analyzed. IMTXTriA33αS was able to release the characteristic α-fragment in a reticulocyte assay by cleaving the rRNA sarcin–ricin loop (SRL). As shown in Figure 4a, the incubation of ribosomes with the trimeric IMTX (2, 6, or 12 pmol) produced the characteristic liberation of the α-fragment, in a similar way to the free α-sarcin (6 pmol used). This confirms that α-sarcin ribonucleolytic activity was preserved in IMXTriA33αS.

#### 2.3.2. Functionality of the Targeting Domain

The ability of the targeting domain to bind GPA33 was analyzed by flow cytometry of GPA33-positive SW1222 cells treated with different concentrations of IMTXTriA33αS. The study was performed using both the monomeric IMTXA33αS and the trimeric IMTXTriA33αS to detect any increase in the binding of the IMTX. The results showed that IMTXTriA33αS binds specifically to the GPA33-positive cells, showing higher binding rates at two different concentrations (0.1 nm and 0.01 nM) than the non-trimeric form of the IMTX (Figure 4b), indicating an increase in the binding activity of IMTXTriA33αS.

#### 2.3.3. Binding and Internalization of IMTXTriA33As

The efficacy of the internalization of IMTXTriA33αS after binding to GPA33 was analyzed on SW1222 cells through fluorescence confocal microscopy. Cells were incubated with IMTXTriA33αS-Alexa555 and the fluorescence signal was analyzed at different times. As shown in Figure 4c, the greatest amount of trimeric IMTX was bound to the cell membrane at 20 min of incubation. After an hour of incubation, there was a significant decrease in the trimeric IMTX in the cell membrane due to the internalization of the IMTX to the cytoplasm.

#### 2.3.4. In Vitro Cytotoxicity Characterization

**Figure 5:**
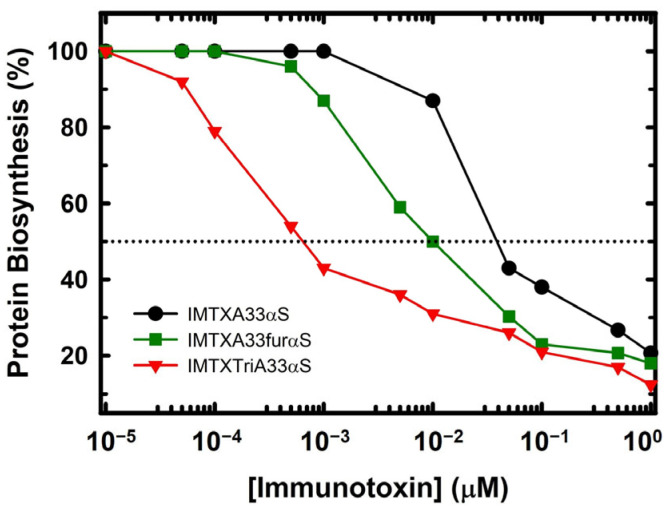
In vitro cytotoxicity characterization. Protein biosynthesis inhibition assay: GPA33-positive cells SW1222 were treated for 72 h with IMTXTriA33αS (red triangles), IMTXA33furαS (green squares), or IMTXA33αS (black circles). Biosynthesis inhibition was measured in terms of L-[4,5-^3^H]-Leucine incorporation. Protein concentrations are expressed in molar units. Measurements were taken and plotted (mean ± SD) and referred to untreated controls. In all cases, triplicate samples were used. Dotted line corresponds to 50% protein biosynthesis inhibition.

The inhibition of protein biosynthesis was analyzed to determine the potential increase in the cytotoxic efficacy of the IMTXTriA33αS compared to its monomeric counterpart, IMTXA33αS. As shown in Figure 5, the cytotoxic activity of IMTXTriA33αS was significantly greater than its monomeric counterpart. The IC_50_ value of IMTXTriA33αS was around 750 pM, whereas the monomeric one was around 30 nM at the same monomer concentration, and even compared to the previously described optimized IMTXA33furaαS [46], proving its therapeutic potential.

This increased activity is coherent with the fact that trimeric IMTXs have higher binding to the cells than the monomeric ones, with a higher internalization rate, and therefore a higher toxic domain concentration on the cytoplasm. Furthermore, IC_50_ decreases significantly more than that observed between the other trimeric immunotoxins described and their corresponding monomeric versions (Table 1). Thus, IMTXTriA33αS exhibits the greatest increase in cytotoxic effectiveness due to its trimeric format.

#### 2.3.5. In Vivo Antitumoral Activity

Finally, the in vivo antitumor activity of the trimeric IMTX was assayed to determine if IMTXTriA33αS reduced tumor growth in BALB/c nude mice. It should be noted that previous studies had already demonstrated the absence of an antitumoral effect in vivo of the target domain scFvA33 or α-sarcin alone [45]. In this work, four experimental groups were used (*n* = 5), including PBS control and three IMTX doses: 25 μg, 50 μg, and 100 μg.

**Figure 6:**
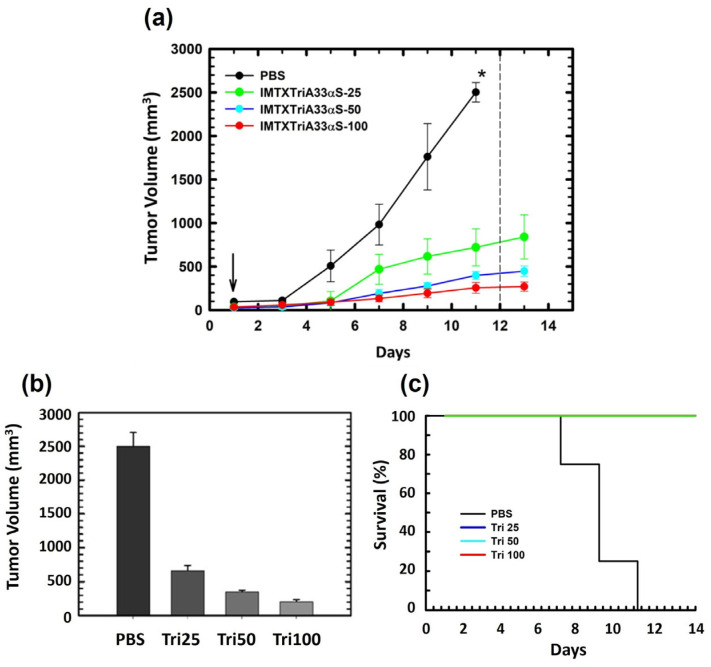
In vivo antitumoral activity. (**a**) Time course of tumor volume progression of SW1222-derived xenografts. Mice were non-treated (PBS) or treated with three different doses (25, 50, or 100 μg) of IMTXTriA33αS per injection. The arrow indicates the beginning of treatment. Doses were given every 48 h. The dashed line indicates the end of treatment. The * indicates that, because of the tumor growth, animals had to be sacrified. (**b**) Weight measurements of excised tumors of non-treated (PBS) or after in vivo treatment with the different doses of IMTXTriA33αS. (**c**) Kaplan–Meier survival curves: The Kaplan–Meier plots show the time to the experimental endpoint (once tumor volume reached 2000 mm^3^) of the in vivo assay. Last data used correspond to day 14 since the beginning of treatment. Plots: PBS (black); Tri-25 (dark blue); Tri-50 (light blue); and Tri-100 (red). The overlap of the latter three makes it appear in green.

Figure 6a shows a very significant antitumor effect of the trimeric IMTX on the reduction in tumor growth, in a dose-dependent manner. At the end of the treatment, the tumor volume of mice treated with 25, 50, and 100 μg of trimeric IMTXTriA33αS were five-, eight-, and ten-fold lower, respectively, than the controls (Figure 6b). Consequently, mice treated with IMTXTriA33αS showed a higher survival rate after 14 days of treatment, as shown in the Kaplan–Meier curves (Figure 6c). None of the mice in the groups treated with IMTX had to be sacrificed, according to the experimental endpoint established.

These results demonstrated an optimization of the antitumor effect of the IMTXTriA33αS, due to the trimeric design being an advance for the potential therapeutic application of IMTXs. In this sense, the reduction is significantly greater than that described previously for the same experimental groups treated with the monomeric form, IMTX33αS [45], with a final tumor volume decrease of 11.6-fold when the highest amount of IMTX (100 µg) was administered, or with IMTXTRICEAαS (Table 2).

## 3. Discussion

The design of the optimized IMTXs for antitumor in vivo treatment has to overcome the issues that nowadays make their therapeutic use difficult, such as a low distribution rate, low tumor penetrability, and unspecific toxicity [4,47]. Some attempts to improve the efficacy of the IMTX designs include selecting the appropriate antigens to target and facilitating the cytosol delivery, either by incorporating a protease cleavage site [46], or by the separation of both domains due to the instability of the target domain in the acidic environment of the lysosome, leading to the translocation of the toxic domain to the cytosol [48]. In addition, the incorporation of different domains that bind to human serum albumin [49] or PEGylation could increase the half-life and reduce the immunogenicity of the therapeutic agent.

In this way, α-sarcin has demonstrated high potential as a toxic domain, due to its specific ribonucleolytic activity, high stability, and low immunogenicity. Fusion of this toxin to a scFv fragment allows the specific delivery of the toxin and its preferential accumulation at the tumor site [44,46]. Our group has tested different designs of IMTXs based on α-sarcin, being IMTXA33αS [44] one of the most promising examples, based on the specific targeting domain scFvA33, which is already humanized. This IMTX has an antitumor effect in vivo when used intravenously, showing an inhibition of tumor growth 6-fold less than the control [45]. Based on these promising results, and taking advantage of the knowledge on trimeric designs [11,12,39,50], we decided to generate the trimeric anti-GPA33 IMTX based on α-sarcin, IMTXTriA33αS, with three toxic domains and three targeting domains per molecule.

The trimeric IMTX was produced and purified with a high yield rate from *Pichia pastoris*, and this structural characterization showed a secondary structure and molecular weight according to a trimeric globular β-sheet rich protein. The functional in vitro characterization showed that the construct maintained the specific binding to its antigen (GPA33), the internalization capability in target cells, and the ribonucleolytic activity of the toxic domain after 72 h in physiological conditions. IMTXTriA33αS showed an increased cytotoxic efficacy in comparison to its monomeric form, with picomolar IC_50_ ranks (750 pM) versus 30 nM (*p* < 0.01), respectively. This result demonstrates the higher performance of trimeric designs, without altering the specificity binding to the antigen, as shown before with other trimeric IMTXs against CEA or EGFR (Table 1) [11,12]. Previously, we have demonstrated the lack of scFvA33 binding to GPA33-negative cells [51]. The significant increment of in vitro cytotoxic activity of IMTXTriA33αS is explained by the trivalent format of the IMTX: higher avidity for its antigen (GPA33), lower dissociation rate than the monomeric IMTX [11,36,52], and triple charge of toxic payload per molecule, probably promoting a major liberation rate of the toxic domain into the cells after internalization. These results suggest a synergic effect due to the trimerization of IMTX, but more kinetic internalization assays and avidity quantification for comparing monomeric and trimeric IMTX should be performed.

The IMTXTriA33αS caused the inhibition of the tumor growth in vivo, using nude mice with subcutaneous SW1222 xenografts cells, without unspecific toxic effect, according to the evolution of the weight, size, and physical appearance of the mice. Treated mice showed decreased tumor growth till the end of the experiment, in comparison with the control mice, which were sacrified once tumor volume reached 2000 mm^3^. The absence of an antitumoral effect in vivo of the target domain scFvA33 or α-sarcin alone had been previously described [45].

This result suggests an improvement on biodistribution, half-life, and tumor targeting thanks to the trimeric design compared to its monomeric counterpart, IMTXA33αS, as described for other trimeric immunoconjugates [39,53]. Therefore, IMTXTriA33αS involves an optimization of the monomeric variant, based on this improved tumor location and toxic payload reached on the cells. These results, in combination with the ones obtained for IMTXTriCEAαS [11] and V_HH_TriEGFRαS [12], the other trimeric IMTXs previously described, support the use of trimeric designs as scaffolds and tools for the novel and ideal optimization of antibodies and bioactive molecules against cancer [33,34,39].

A promising field of study is the use of trimeric designs with different targeting domains on the same IMTX molecule, increasing therefore its specificity and avidity to tumor cells, avoiding tumor evasion due to the loss of a single-targeted tumor-associated antigen [54], aside from addressing the immunogenicity, safety, and pharmacokinetics of these new therapeutic trimeric designs [55]. The potential application of trimeric IMTXs in vivo will require further studies to assess the treatment’s long-term effects on tumor development and the adverse effects on healthy tissues and organs.

## 4. Materials and Methods

### 4.1. Plasmid Design

To obtain the expression vector for IMTXTriA33αS, we used the expression vector pPICZαAIMTXA33αS containing the sequence of the monomeric IMTX [44], adding the codification sequence of the trimerization domain TIE^XVIII^. This sequence was amplified by PCR from the plasmid pPICZαAIMTXTriCEAαS [11]. From this plasmid, we have obtained the L21-TIE^XVIII^-L21 fragment, followed by a ligation, after *NotI* digestion, to the pPICZαAIMTXA33αS. Once the correct sequence was confirmed through Sanger sequencing by the Genomic Unit at the Universidad Complutense de Madrid, plasmid digestion with *PmeI* was carried out. The linearized plasmid was used for *P. pastoris* Bg11 electroporation, using a Bio-Rad Gene Pulser device (Bio-Rad, Hercules, CA, USA).

### 4.2. Protein Production and Purification

IMTXTriA33αS was expressed in *Pichia pastoris* and purified from the extracellular media by Ni-NTA affinity chromatography, dialysis, and exclusion chromatography, as described previously [44]. Aliquots from the different purification steps were analyzed through SDS-PAGE followed by Coomassie Blue staining, or by Western blotting using a rabbit anti-α-sarcin serum.

Briefly, electrocompetent *Pichia pastoris* Bg11 cells were electroporated with 10 μg of the linearized plasmid. The electroporated cell mixture was seeded in YPDS plates containing different zeocin concentrations (100 to 750 μg/mL). Multiple clones were selected to test the optimal conditions for their expression. In the first step, Dot blot screening using an anti-α-sarcin antibody was carried out. Two colonies, 3-Zeo^750^ and 7-Zeo^400^, were selected to perform a low scale production of the colonies, modifying the induction time (24–72 h) and temperature (15–25 °C). Protein expression was analyzed using 0.1% (*w*/*v*) sodium dodecyl sulfate (SDS)–15% polyacrylamide gel electrophoresis (PAGE) and Western blot using an anti-α-sarcin antibody.

Large-scale expression was carried out with the selected yeast clone using 2 L of BMGY medium culture, using baffled Erlenmeyer flasks, at 30 °C with vigorous agitation. Cells were harvested by centrifugation and resuspended in 1 L of BMMY for induction for 24 h. The extracellular medium was dialyzed two times against 50 mM sodium phosphate, 0.1 M NaCl, and pH 7.5 buffer.

Purification was carried out using immobilized metal affinity chromatography with a Ni^2+^-NTA agarose column (GE Healthcare, Uppsala, Sweden). The dialyzed extracellular medium was loaded into the column at a flow rate of 1 mL/min. Then, the column was washed with 50 mM sodium phosphate, 0.1 M NaCl, and pH 7.5 buffer, and with 20 mM imidazole in phosphate buffer. The IMTXTriA33αS was eluted using the same buffer containing 250 mM imidazole. Fractions containing the IMTXTriA33αS were pooled and dialyzed against a sodium phosphate buffer to remove the imidazole. The different fractions were analyzed by SDS-PAGE followed by Coomassie Blue staining or Western blot using an anti-α-sarcin polyclonal antibody.

### 4.3. Structural Characterization

Structural and functional characterization was performed as described previously [44,46], before any in vivo assay. A new step of purification and characterization was used, using an Aktä Purifier FPLC (Fast Liquid Chromatography) system (GE Healthcare Lifescience, Lifescience, Marlborough, MA, USA) coupled to a Superdex 200 10/300GL column to determine the exact molecular weight and the oligomerization state of the trimeric immunotoxin. FPLC chromatography was performed with the IMTX dissolved in PBS, with a constant flow of 1 mL/min. Far-UV circular dichroism (CD) analysis was obtained through a JASCO J-175 spectropolarimeter (Jasco Analitica, Madrid, Spain) at a scanning speed of 40 nm/min. IMTXTriA33αS was dissolved in phosphate buffer NaCl 0.5 M at a final concentration of 0.15 mg/mL. At least six spectra were obtained and averaged to obtain the final result. The purified protein was lyophilized and stored at −80 °C if needed.

### 4.4. Ribonucleolytic Activity Assay

The specific ribonucleolytic activity of α-sarcin, which is included as the toxic domain of IMTXTriA33αS, was analyzed as previously described [56,57], based on the detection of the 400 nt rRNA fragment (α-fragment) released from ribosomes from a reticulocyte lysate. Briefly, aliquots of 50 µL of the reticulocyte assay were incubated with the different IMTXs/conditions for 15 min at room temperature (RT). The reaction was stopped by the addition of Tris 50 mM, SDS 5% pH 7.4 buffer. Resulting RNA phenol/chloroform extraction was carried out, followed by RNA precipitation with isopropanol. The presence of the α-fragment was detected through a 2% agarose 16% paraformaldehyde gel, followed by ethidium bromide staining. Images were obtained with a Universal hood II transilluminator (Bio-Rad, Hercules, CA, USA).

### 4.5. Cell Line Cultures

As a GPA33-positive cell, the human carcinoma cell line SW1222 was used, both for in vitro and in vivo assays. These cells have a spontaneous differentiation, and were obtained from Dr. Carl Batt, under the collaboration of Ludwig Cancer Institute. No GPA33-negative cells were used due to the previous characterization of the monomer in terms of specificity [51]. Cells were grown on DMEM (Dulbecco’s Modified Eagle’s Medium), containing 50 μg/mL of streptomycin, 10% of fetal bovine serum (FBS), 50 U/mL of penicillin, and 300 μg/mL of L-glutamine. Incubation was performed in humified atmosphere, with 5% CO_2_ and at 37 °C. Routine harvesting and propagation were performed by trypsinization.

### 4.6. Flow Cytometry Assays

Cells were harvested via trypsinization, centrifugation at 1500 rpm, 10 min, 4 °C, and resuspension on culture media with a cellular density of 5 × 10^5^ cells/mL. Each cytometer tube was filled with the corresponding volume to reach 1 × 10^5^–5 × 10^5^ cells/tube. Tubes were centrifuged at 1500 rpm, 10 min, 4 °C, and cells were resuspended on PBS-BSA 1%, followed by addition of the monomeric or trimeric IMTX at the different concentrations to study: 0.1 nM and/or 0.01 nM, and incubation for 1 h at RT. The centrifugation and washing steps were repeated, and cells were resuspended in PBS-BSA 1% containing Histag-488/GAR-448, being incubated for 1 h in darkness at RT. Finally, cells were once again washed and centrifugated, and pellets were resuspended in 300 μL of buffer and stored at 4 °C in darkness. Fluorescence was measured using a FACScan (Becton Dickinson, Franklin Lakes, NJ, USA) and analyzed using the WinMDI v 2.9 software.

### 4.7. Alexa 555 Labeling of IMTXTriA33αS

Alexa Fluor 555 Protein Labeling Kit (Invitrogen, Carlsbad, CA, USA) was used for labeling IMTXTriA33αS, incubating 100 μg of IMTX with 44.6 nmol of Alexa 555 for 15 min, on bicarbonate 0.1 M, pH 8.3. The conjugate was purified by affinity chromatography, and the labeling grade was determined based on absorbance at 280 nm and 555 nm.

### 4.8. Immunofluorescence Microscopy

The 80 × 10^3^–100 × 10^3^ cells were seeded and grown for one night in culture conditions previously described in this work, but with 20% of FBS. Once cells reached 50–70% of confluence, media were eliminated and the IMTX was added. In the case of binding studies, cells were incubated with the IMTX for 30 min with new media. For the internalization study, proteins were added with new media and retired after 30 min of incubation, adding new media and incubating the cells at 37 °C for 1 h. Next, cells were washed three times with PBS. Cells were fixed with paraformaldehyde (PFA) 3% for 15 min at room temperature (RT). Three new washing steps were carried out, and PFA was quenched by adding 50 mM ammonium chloride on PBS, for 15 min at RT. Another step of washing was performed with PBS-BSA 1% and the cells were incubated with primary Ab, CD44, or anti-α-sarcin, for 15 min or 3 h, respectively, followed by incubation with GAR-Alexa488 for 1 h. Finally, cells were fixed to the cover slide with Prolong Gold (Invitrogen) containing DAPI. Samples were stored at 4 °C in darkness until their utilization.

### 4.9. Protein Biosynthesis Inhibition Assay

To analyze the cytotoxicity of ribotoxins, protein biosynthesis inhibition assay was carried out as described [58]. Briefly, cells were seeded in 96-well plates at a density of 1 × 10^4^ cells/well in culture medium for 24 h. Then, different concentrations of each IMTX were added in 200 µL of free-FBS fresh medium. After 72 h of culture incubation, the medium was replaced with fresh medium containing 1 mCi per well of L-[4-C-3H]-Leucine (166 Ci/mmol; GE Healthcare, Fairfield, CT, USA). Then, incubation was carried out for 6 h, followed by fixation of the cells with 5% (*w*/*v*) trichloroacetic acid. After several washing steps with ethanol 70%, pellets were dissolved in 200 µL of 0.1 M NaOH 0.1% SDS buffer. Radioactivity was measured through a Beckman LS3801 liquid scintillation counter. Cytotoxicity was calculated as IC_50_ values, expressed as the percentage of the radioactivity incorporated in the assay. All samples were triplicated.

### 4.10. In Vivo Antitumor Assay

Balb/c nude male mice with 7 weeks of life were obtained from Harlan Laboratories S.A. (Barcelona, Spain) to evaluate in vivo effect of IMTXTriA33αS against induced xenograft of colorectal cancer. The caring of the mice was carried out in the Animal Facilities of the UCM (Complutense University of Madrid) and the Centro de Investigaciones Biológicas of the Consejo Superior de Investigaciones Científicas (CIB-CSIC). Mice were given an adaptation period of 7 days with free access to water and food, prior to the inoculation of tumor cells. Cells were harvested and washed three times with PBS (Phosphate-Buffered Saline) and prepared for the injection of 2 × 10^8^ cells on 200 μL of PBS-Matrigel (BD Biosciences, San Jose, CA, USA) (1:1) to each mouse. Inoculation of tumor cells was performed subcutaneously on the right side of the mice, and tumor growth was followed until they reached a size between 50 and 100 mm^3^, when treatment started. Mice (*n* = 5) were distributed into 3 different experimental groups randomly, for the study with 3 different concentrations of IMTX: 25 μg, 50 μg, or 100 μg, each one with a control group. The injection of the IMTX or PBS was intraperitoneal, on a total volume of 100 μL. Seven doses of the different treatments were given every 48 h. Tumor growth was followed by tumor external size measurement every two days, and mice were weighed throughout the experiment. Once the study was finished, mice were sacrificed. All animal procedures were performed with the approval of the Complutense University Animal Experimentation Committee and the Community of Madrid (PROEX298/15), according to the official European regulations.

### 4.11. Statistical Analysis

ANOVA with a post hoc analysis via the Student–Newman–Keuls test was used within each test to compare the results obtained with the different concentrations of the immunotoxins in the different assays. All values are expressed as arithmetic means ± standard error of the media (sem). Differences among experimental groups were considered statistically significant at *p* < 0.05.

## 5. Conclusions

The results included in this work confirm the great potential of trimeric IMTXs based on ribotoxins and represent a step forward in the development of new therapeutic strategies. The trimeric IMTX described in this work enhances the overall antitumor efficacy due to its increased avidity and toxic payload. Its modular structure allows for the development of new IMTX designs that combine multimeric formats and different specificities, leading to new therapeutic features for cancer and other diseases, such as allergies.

## Figures and Tables

**Table 1:** In vitro cytotoxicity comparison of different trimeric IMTXs based on α-sarcin. IC_50_ values (nM) obtained after 72 h incubation with their target cells (SW1222 or A431). In brackets, IC_50_ values of each of their monomeric counterparts. * Data described in [11]; ** described in [12].

	IC_50_ (nM)	IC_50_ Decrease (Nº Folds) Trimeric vs Monomeric IMTX	*p* Value
IMTXTriA33αS	0.75 (30.0)	40	<0.001
IMTXTriCEAαS *	6.0 (60.0)	10	<0.001
VHHTriEGFRαS **	10 (300.0)	30	<0.001

**Table 2:** Statistical analysis of IMTXTriA33αS-treated tumors vs. vehicle-treated tumors at the end of the treatment (day 13) and IMTXTriA33αS vs. IMTX33αS [45]. * Data previously obtained for IMTXTRICEAαS-treated groups [11] or ** monomeric IMTXTA33αS-treated groups [45] are shown for comparison.

Group	Tumor Volume (mm^3^) Day 13	*p* Value	Tumor Volumen Decrease (Nº folds)	Comparison	*p* Value
PBS	2970	-			
TRIA33-25	839.1 ± 246.4	<0.001	3.5		
TRIA33-50	442 ± 61.81	<0.001	6.7	A33-50 ** vs TRIA33-50	<0.01
TRIA33-100	256.6 ± 60.1	<0.001	11.6	A33-100 ** vs TRIA33-100	<0.01
PBS *	1960.4 ± 403.8	-			
TRICEA-25 *	414.9 ± 75.3	<0.001	4.7		
TRICEA-50 *	300.6 ± 52.5	<0.001	6.5		

## Data Availability

The original contributions presented in this study are included in the article/Supplementary Materials. Further inquiries can be directed to the corresponding author.

## Supplementary Materials

The following supporting information can be downloaded at https://www.mdpi.com/article/10.3390/ijms27020764/s1.
