# Supplementary material for: GPA33-Targeted Trimeric Immunotoxin Exhibits Enhanced Antitumor Activity in Human Colorectal Cancer Xenografts
Source: Int J Mol Sci. 2026 Jan 12;27(2):764. doi: 10.3390/ijms27020764 (PMC12841415; doi:10.3390/ijms27020764)
Supplement: Supplementary file 1 [file ijms-27-00764-s001.zip › ijms-4069247-supplementary.pdf]

GPA33-targeted trimeric Immunotoxin exhibits enhanced Antitumor Activity in Human colorectal cancer Xenografts

Ruiz-de-la-Herrán, J

Narbona, J.

Gordo, R.G.

Sanz, L

Lacadena, J.

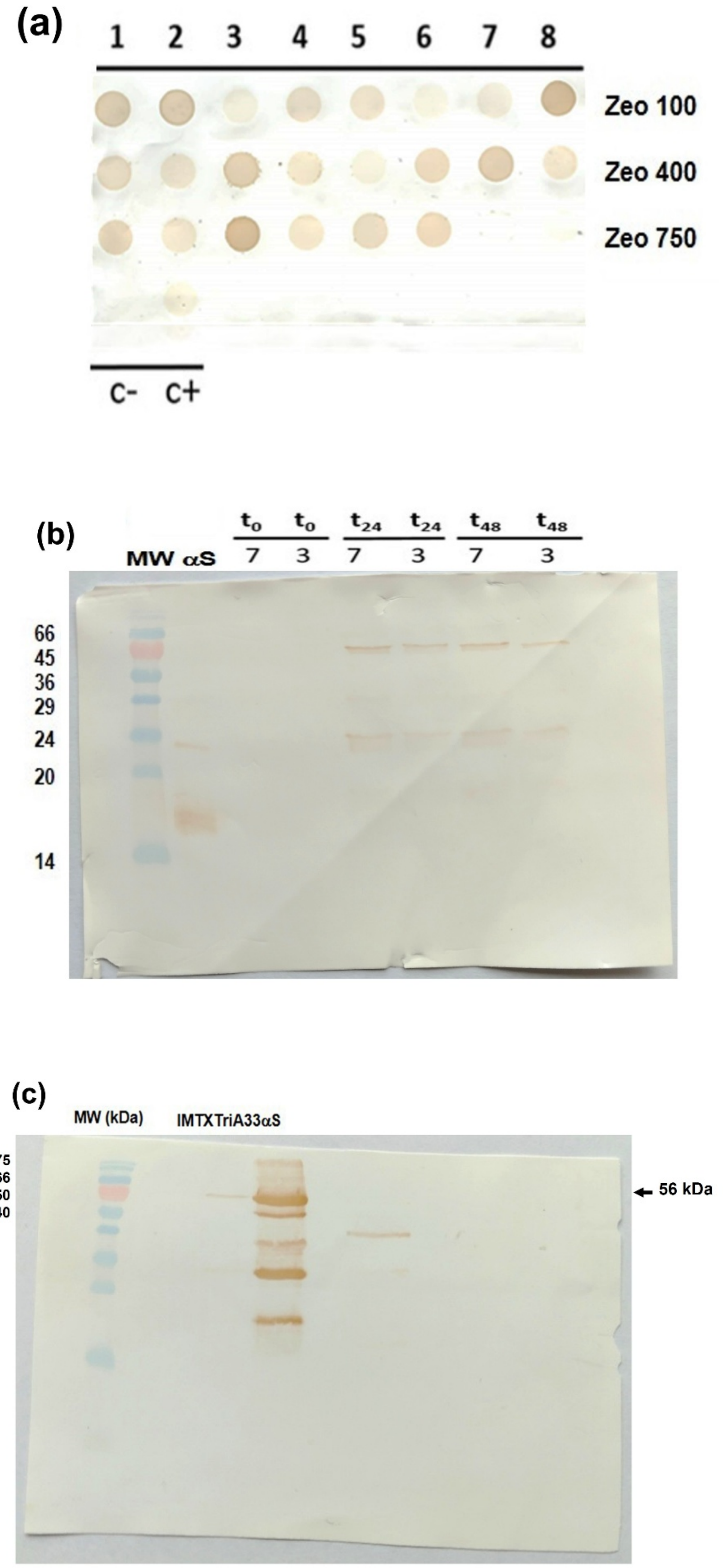
(a)
1 2 3 4 5 6 7 8
Zeo 100
Zeo 400
Zeo 750
C- C+
(b)
MW αS
t0 t0 t24 t24 t48 t48
7 3 7 3 7 3
66
45
36
29
24
20
14
(c)
MW (kDa)
IMTXTriA33αS
75
66
50
40
56 kDa

Figure S1

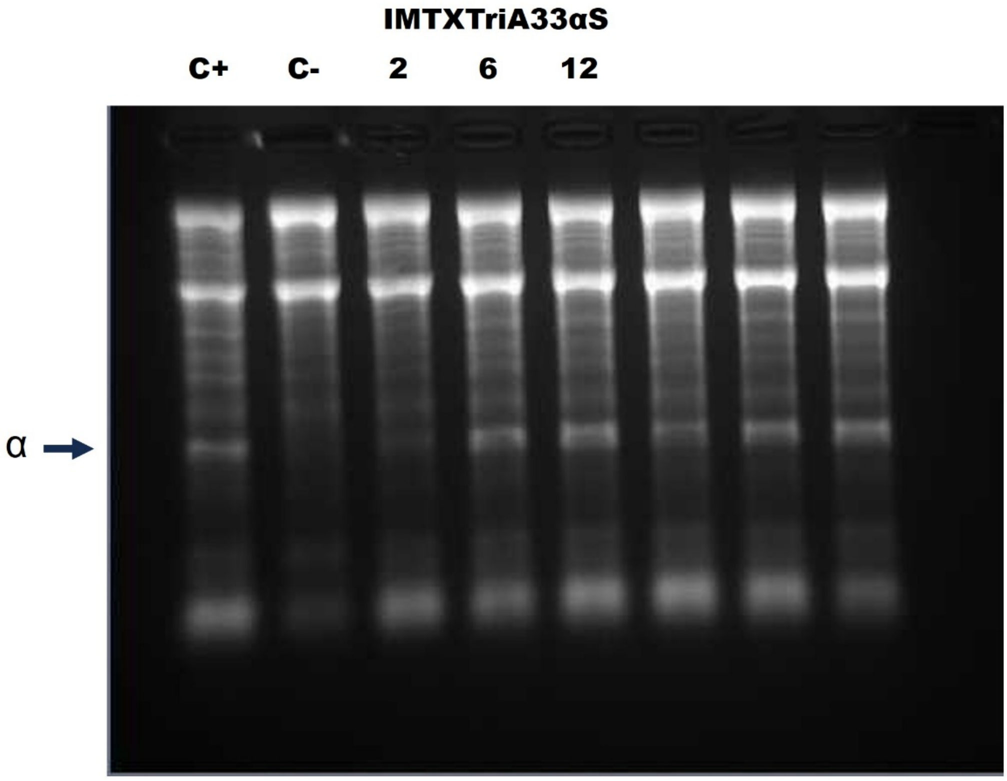

Figure S2

Supplementary Figures Legends

Figure-S1. Production and purification assays. (a) Multiple colony expression analysis by Dot blot. The numbers indicate the colony number among those tested for each zeocin concentration. C- and C+, located in the 4th row, columns 1 and 2, correspond to the negative control (non-transformed colonies) and positive control (IMTXA33αS-producing colonies), respectively. Original dot blot from Figure 2a is shown, with brightness and contrast adjusted to reveal the edges of the membrane. (b) Western blot analysis of expression assays of 3-Zeo750 and 7-Zeo400 colonies, pre-selected by Dot blot, at different induction times (t0, t24 and t48). MW corresponds to the molecular mass pattern. The second lane corresponds to the α-sarcin positive control. (c) *Western Blot* analysis of purified IMTXTriA33αS using rabbit anti-α-sarcin serum. MW corresponds to prestained Bio-Rad Precision Plus protein molecular weight standards. Original full-length western blots from Figure 2b and 2e are presented. Images corresponding to blots were acquired and analyzed using ChemiDoc-It (UVP) and VisionWorks LS, respectively.

Figure-S2. **Functional characterization of the ribonucleolytic activity.** The agarose gel represents the ribonucleolytic activity of α-sarcin in IMTXTriA33αS. The arrow indicates the presence of the α-fragment. 2, 6 and 12 pmoles of IMTXTriA33αS were tested. C+ represents 6 pmoles of fungal wild-type α-sarcin, and in C- the protein sample was replaced by buffer. Original full-length gel from Figure 4a is presented. Gel image was acquired and analyzed using the Gel Doc XR Imaging System and Quantity One 1-D analysis software (BioRad).
